# Proneness to high blood lipid-related indices in female smokers

**DOI:** 10.1186/s12944-019-1050-3

**Published:** 2019-05-13

**Authors:** Yuri Chimura, Takashi Daimon, Ichiro Wakabayashi

**Affiliations:** 1 Department of Environmental and Preventive Medicine, Hyogo College of Medicine, Mukogawa-cho 1-1, Nishinomiya, Hyogo 663-8501 Japan; 2 Division of Biostatistics, Hyogo College of Medicine, Nishinomiya, Hyogo 663-8501 Japan

**Keywords:** Alcohol, Blood lipids, Cardiovascular disease, Smoking, Women

## Abstract

**Background:**

Smoking is a major risk factor for dyslipidemia. However, it remains to be clarified whether light smoking in Asian women affects lipid profiles and lipid-related indices. The aim of this study was to determine the relationships between lipid-related indices and smoking in Japanese women. Alcohol drinking influences blood lipid levels and is a potent confounder for the relationship between smoking and blood lipids. Thus, analysis for the relationships between smoking and blood lipid-related indices was also performed after stratification of drinking status.

**Methods:**

The participants were 18,793 Japanese women aged 35–70 years. A cross-sectional study was performed using a local population-based database. The relationships of smoking with each index were investigated by using analysis of covariance and logistic regression analysis with adjustment for age and other lifestyle factors such as alcohol drinking and regular exercise.

**Results:**

In multivariate logistic regression analysis, odds ratios of smokers vs. nonsmokers for high ratio of LDL cholesterol to HDL cholesterol (LDL-C/HDL-C), high ratio of triglycerides to HDL cholesterol (TG/HDL-C), high lipid accumulation product (LAP) and high cardio metabolic index (CMI) were significantly higher than the reference level of 1.00 in overall participants (2.17 [1.78–2.66], 1.70 [1.47–1.97], 1.17 [1.08–1.27] and 1.41 [1.30–1.53], respectively), nondrinking participants (2.29 [1.80–2.91], 1.68 [1.39–2.02], 1.21 [1.08–1.36] and 1.46 [1.30–1.63], respectively), and drinking participants (1.96 [1.35–2.85], 1.76 [1.39–2.21], 1.13 [1.01–1.27] and 1.38 [1.22–1.55], respectively). In overall participants, nondrinking participants, and drinking participants, LDL-C/HDL-C, TG/HDL-C, LAP and CMI were significantly higher in smokers than in nonsmokers. In nondrinking participants, triglycerides and LDL cholesterol were significantly higher in smokers than in nonsmokers, while the ratio of waist circumference to height and HDL cholesterol were significantly lower in smokers than in nonsmokers.

**Conclusion:**

In women, all of the four lipid-related indices tested were higher in smokers than in nonsmokers, and these associations were independent of alcohol drinking. The high levels of the lipid-related indices in smokers result from the detrimental effects of smoking on levels of blood lipids such as triglycerides, HDL cholesterol and LDL cholesterol.

## Background

Dyslipidemia is a major risk factor for cardiovascular disease [1]. Lipid-related indices, including the ratio of LDL cholesterol to HDL cholesterol (LDL-C/HDL-C), ratio of triglycerides to HDL cholesterol (TG/HDL-C), lipid accumulation product (LAP) and cardio metabolic index (CMI), have been proposed to be useful predictors for the risk of cardiovascular disease. LDL-C/HDL-C is a classical atherogenic index that predicts the risk of ischemic heart disease [2]. TG/HDL-C has been shown to be a better predictor of cardiovascular disease than LDL-C/HDL-C [3, 4] and to reflect a strong atherogenic molecule, small dense LDL [5]. LAP, calculated by using triglycerides and waist circumference [6], has been reported to be a better predictor of diabetes and cardiovascular disease than body mass index [7–9]. More recently, CMI, defined as the product of TG/HDL-C and the ratio of waist circumference to height (WHtR) [10], has been proposed to be a discriminator for diabetes and has been shown to be associated with the degree of atherosclerotic progression in patients with peripheral arterial disease [11].

Habitual smoking is a potent risk factor for cardiovascular diseases, including ischemic heart disease, stroke, peripheral arterial disease, and aortic abdominal aneurysm [12]. There are different mechanisms for deterioration of cardiovascular health by smoking: smoking induces dyslipidemia, increases blood coagulability, and causes oxidative stress [13]. The results of a previous meta-analysis showed that HDL cholesterol and triglycerides were significantly lower (− 5.7%) and higher (9.1%), respectively, in smokers than in nonsmokers, while LDL cholesterol was not significantly different between nonsmokers and smokers (1.7%) [14]. These differences in blood lipid levels in smokers and nonsmokers are thought to reflect smoking-induced changes in lipid metabolism: smokers have been reported to have lower activities of lipoprotein lipase (LPL) and lecithin cholesterol acyltransferase (LCAT) and a higher activity of cholesterol ester transfer protein (CETP) than those in nonsmokers [15, 16]. A randomized clinical trial showed that one-year smoking cessation resulted in a significant increase in HDL cholesterol but no change in LDL cholesterol [17].

According to the results of the national survey in Japan [18], the male smoking rate declined considerably during the past quarter of a century (53.1% in 1990 vs. 32.2% in 2014), while the female smoking rate did not change greatly (9.7% in 1990 vs. 8.5% in 2014). Moreover, a recent systematic review and meta-analysis study has shown a greater effect of smoking on ischemic heart disease in women than in men [19]. Women were also reported to have a larger increase in HDL cholesterol after smoking cessation than men [17]. Thus, cessation of smoking, a modifiable lifestyle, in women is an important issue in public health for prevention of cardiovascular disease. Although smoking is related to dyslipidemia such as hypo-HDL-cholesterolemia and hyper-triglyceridemia [14], it remains to be clarified whether and how smoking affects lipid-related indices in women.

The purpose of this study was to determine the relationships of smoking with four lipid-related indices (LDL-C/HDL-C, TG/HDL-C, LAP and CMI) and the variables comprising the indices (waist circumference, WHtR, triglycerides, HDL cholesterol and LDL cholesterol) in women. There is a strong positive relationship between alcohol consumption and smoking: Smokers are more prone to be alcohol drinkers than are nonsmokers [20]. Alcohol drinking potently affects blood lipid levels: Alcohol drinking increases HDL cholesterol and triglycerides and decreases LDL cholesterol [21–23], resulting in changes in blood lipid-related indices. Thus, alcohol drinking is expected to potently confound the relationships of smoking with blood lipid-related indices and, in this study, in order to take the confounding by alcohol into consideration, we analyzed the relationships of smoking with blood lipid-related indices using subgroup analysis by alcohol. Other possible confounders such as age and regular exercise, in addition to alcohol drinking, were used as explanatory variables for adjustment in multivariate analyses (logistic regression analysis and analysis of covaraince).

## Methods

### Participants and study design

The participants were 18,793 Japanese women aged ≥35 and ≤ 70 years who had received periodic health check-up examinations at workplaces in Yamagata Prefecture in Japan. A cross-sectional study was performed using a local population-based database for the above participants. This study was approved by the Hyogo College of Medicine Ethics Committee (No. 3003 in 2018). In a questionnaire at the health checkup, participants were required to identify any conditions for which they were receiving treatment. Those who had been receiving medication therapy for dyslipidemia were excluded from participants of this study. Histories of cigarette smoking, alcohol consumption, regular exercise (almost every day with exercise for 30 min or longer per day), and illness were also surveyed by questionnaires.

In the self-written questionnaire paper, participants were first asked “Are you a habitual cigarette smoker?” Cigarette smokers were defined as participants who had smoked for 6 months or longer and had smoked for the past month or longer. Then the participants who were smokers were further asked “What is your average cigarette consumption per day?”. The response categories for this question were “less than 21 cigarettes per day”, “21 or more and less than 41 cigarettes per day” and “41 or more cigarettes per day”. In this study, only two categories of smoking, nonsmokers and smokers, were used for analysis because the percentage of heavy smokers who smoked 21 or more cigarettes per day was very low, 0.80% (*n* = 150), in overall participants.

Average alcohol consumption of each participant per week was reported on the questionnaires. Frequency of habitual alcohol drinking was asked in the questionnaire as “How frequently do you drink alcohol?”. Frequency of weekly alcohol drinking was categorized as “every day” (regular drinkers), “sometimes” (occasional drinkers) and “never” (nondrinkers).

### Measurements

Height and body weight were measured with the participants wearing light clothes without a jacket or coat at the health checkup. Waist circumference was measured at the navel level according to the recommendation of the definition of the Japanese Committee for the Diagnostic Criteria of Metabolic Syndrome [24]. Cut-off values of large waist circumference and high WHtR used were 90 cm and 0.5, respectively [24, 25].

Fasted blood was sampled from each participant in the morning, and serum triglyceride, HDL cholesterol and LDL cholesterol levels were measured by enzymatic methods using commercial kits, pureauto S TG-N, cholestest N-HDL and cholestest LDL (Sekisui Medical Co., Ltd., Tokyo, Japan), respectively. The coefficients of variation for reproducibility of measurement were ≤ 3% for triglycerides, ≤ 5% for HDL cholesterol and ≤ 5% for LDL cholesterol. The cut-off values for high triglycerides, low HDL cholesterol and high LDL cholesterol used were 150 mg/dl [26], 50 mg/dl and 140 mg/dl, respectively [26, 27]. The cut-off values for high LDL-C/HDL-C and high TG/HDL-C used were 3.5 and 3.75, respectively. LAP was determined by using triglyceride level (TG) and waist circumference (WC) as follows: LAP = TG (mmol/L) x [WC (cm) – 58] [6]. The cut-off value for high LAP was defined as 21.1 [28]. CMI was calculated as the product of WHtR and TG/HDL-C, and the cut-off value for high CMI used was 0.800 [10].

### Statistical analysis

The purpose of this study was to clarify the relationship between smoking and dyslipidemia in Japanese women using lipid-related variables, especially lipid indices. Thus, the primary analysis was carried out to elucidate the relationship between smoking and dyslipidemia using logistic regression, and the secondary analysis was carried out to confirm the relationship between smoking and level of each variable using analysis of covariance (ANCOVA). Each variable was compared between nonsmokers and smokers in overall participants and in nondrinking and drinking participants separately.

Categorical variables were compared between nonsmokers and smokers using the chi-square test for independence. In logistic regression analysis, odds ratios of smokers vs. nonsmokers for each variable (large waist circumference, high WHtR, high triglycerides, low HDL cholesterol, high LDL cholesterol, high LDL-C/HDL-C, high TG/HDL-C, high LAP or high CMI) were estimated. Interaction analysis was performed using an interaction term consisting of smoking (nonsmokers vs. smokers) and alcohol drinking (nondrinkers vs. drinkers) as an explanatory variable in logistic regression model. As a sensitivity analysis, logistic regression analysis of participants excluding heavy smokers (comparison between light smokers and nonsmokers) was performed since the purpose of this study was to clarify the relationships of light smoking with dyslipidemia in Japanese women. In addition, drinkers were also excluded in the sensitivity analysis since alcohol drinking is a potent confounder for the relationship between smoking and blood lipids [20–23].

For continuous variables, means of each variable were compared between nonsmokers and smokers by using Student’s t test in univariate analysis. In multivariate analysis, the mean levels of each variable were compared by using ANCOVA followed by Student’s t-test after Bonferroni correction. Since triglycerides, TG/HDL-C, LAP and CMI did not show a normal distribution, they were compared non-parametrically by using the Mann-Whitney U test in univariate analysis or were used after logarithmic transformation in ANCOVA. Participants showing waist circumference of 58 cm or smaller (*n* = 66) were excluded from participants in ANCOVA for LAP, because their values for log-transformation were zero or under zero, and thus log-transformed LAP could not be calculated.

Age and a habit of regular exercise were adjusted in ANCOVA and multivariate logistic regression analysis. In addition, a habit of alcohol drinking was added to the explanatory variables in analysis for overall participants (non, occasional and regular drinkers) and drinking participants (occasional and regular drinkers). Each variable in nonsmokers was also compared among nondrinkers, occasional drinkers and regular drinkers by using ANCOVA with adjustment for age and a habit of regular exercise (Table 7).

All *p* values are two-sided and values of *p* less than 0.05 were considered to indicate statistical significance. Statistical analyses were performed using a computer software program (SPSS version 16.0 J for Windows, Chicago IL, USA).

## Results

### Characteristics of participants

Table 1 shows profiles of the participants and results of comparison of each variable between nonsmokers and smokers in univariate analyses. Age was significantly younger in smokers than in nonsmokers. The percentages of occasional drinkers and regular drinkers were significantly higher in smokers than in nonsmokers, while the percentage of participants with a habit of regular exercise was not significantly different in smokers and nonsmokers. In nondrinkers (*n* = 11,408) and drinkers (*n* = 7385), the proportions of smokers were 13.1% (*n* = 1491) and 25.8% (*n* = 1904), respectively: The percentage of smokers was significantly higher (*p* < 0.01) in drinkers than in nondrinkers. Height and waist circumference were slightly but significantly larger and smaller, respectively, in smokers than in nonsmokers. WHtR and the percentage of subjects showing high WHtR were significantly lower in smokers than in nonsmokers.

**Table 1:** Characteristics of participants

Variables	Nonsmokers	Smokers	Overall participants
Number	15,398	3395	18,793
Age (years)	49.1 ± 7.7	45.8 ± 7.3**	48.5 ± 7.7
Drinkers (%)			
Occasional drinkers	28.8	34.8**	29.8
Regular drinkers	6.8	21.3**	9.5
Regular exercise (%)	5.3	5.7	5.4
Height (cm)	156.0 ± 5.6	157.4 ± 5.6**	156.3 ± 5.6
WC (cm)	78.2 ± 9.5	77.1 ± 9.8**	78.0 ± 9.6
WHtR	0.502 ± 0.064	0.490 ± 0.063**	0.500 ± 0.064
Large WC (%)	11.5	10.4	11.3
High WHtR (%)	47.6	39.5**	46.2
Triglycerides (mg/dl)	78 (55, 114)	80 (56, 119)**	78 (55, 115)
HDL-C (mg/dl)	65.5 ± 14.8	64.1 ± 15.5**	65.3 ± 14.9
LDL-C (mg/dl)	115.3 ± 29.7	110.4 ± 31.4**	114.5 ± 30.1
High triglycerides (%)	13.0	14.7**	13.3
Low HDL-C (%)	13.0	16.4**	13.6
High LDL-C (%)	19.8	16.8**	19.3
LDL-C/HDL-C	1.87 ± 0.70	1.86 ± 0.78	1.87 ± 0.72
TG/HDL-C	1.19 (0.78, 1.94)	1.25 (0.81, 2.10)**	1.21 (0.79, 1.97)
LAP	16.6 (9.0, 30.3)	15.6 (8.5, 29.5)*	16.4 (8.9, 30.1)
CMI	0.59 (0.37, 1.02)	0.60 (0.38, 1.06)*	0.59 (0.37, 1.02)
High LDL-C/HDL-C (%)	2.8	4.2**	3.0
High TG/HDL-C (%)	6.3	8.0**	6.6
High LAP (%)	39.3	37.4*	39.0
High CMI (%)	35.1	36.5	35.3

Shown are numbers, frequencies, means ± standard deviations, and medians with 25 and 75 percentile values. WC, waist circumference; WHtR, ratio of waist circumference to height; HDL-C, HDL cholesterol; LDL-C, LDL cholesterol; LDL-C/HDL-C, ratio of LDL cholesterol to HDL cholesterol; TG/HDL-C, ratio of triglycerides to HDL cholesterol. Asterisks denote significant differences from nonsmokers (*, *p* < 0.05; **, *p* < 0.01)

Triglycerides were significantly higher and HDL cholesterol and LDL cholesterol were significantly lower in smokers than in nonsmokers. The percentages of participants showing high triglycerides and low HDL cholesterol were significantly higher in smokers than in nonsmokers, while the percentage of participants showing high LDL cholesterol was significantly lower in smokers than in nonsmokers.

TG/HDL-C and CMI were slightly but significantly higher in smokers than in nonsmokers, while LAP was slightly but significantly lower in smokers than in nonsmokers, and LDL-C/HDL-C was not significantly different in nonsmokers and smokers. The percentages of participants showing high LDL-C/HDL-C and high TG/HDL-C were significantly higher in smokers than in nonsmokers, while the percentage of participants showing high LAP was slightly but significantly lower in smokers than in nonsmokers, and the percentage of participants showing high CMI was not significantly different in nonsmokers and smokers.

### Odds ratios of smokers vs. nonsmokers for abnormal levels of each variable comprising lipid-related indices

The relationships of smoking with abnormal levels of each variable comprising lipid-related indices were investigated by using logistic regression analysis (Table 2). The odds ratios of smokers vs. nonsmokers for high WHtR were slightly but significantly or marginally significantly lower than the reference level of 1.00 in overall participants, nondrinking participants and drinking participants, while in all of these participant groups, the odds ratios of smokers vs. nonsmokers for large waist circumference were not significantly different from the reference level. In overall participants, nondrinking participants and drinking participants, the odds ratios of smokers vs. nonsmokers for high triglycerides and low HDL cholesterol were significantly higher than the reference level. The adjusted odds ratios for high LDL cholesterol were significantly higher than the reference level in nondrinking participants and overall participants, while the adjusted odds ratio was not significantly different from the reference level in drinking participants.

**Table 2:** Odds ratios of smokers vs. nonsmokers for abnormal levels of each variable comprising lipid-related indices in nondrinking participants, drinking participants and overall participants

Variables	Nondrinkers (*n* = 11,408)	Drinkers (*n* = 7385)	Overall participants (*n* = 18,793)
Large WC			
Crude	0.97 (0.82–1.15)	0.88 (0.73–1.05)	0.89 (0.79–1.00)
Adjusted	1.05 (0.89–1.25)	0.99 (0.82–1.19)	1.02 (0.90–1.15)
High WHtR			
Crude	0.74 (0.67–0.83)**	0.75 (0.67–0.83)**	0.72 (0.67–0.77)**
Adjusted	0.90 (0.80–1.01)#	0.90 (0.80–1.01)##	0.90 (0.83–0.97)**
High triglycerides			
Crude	1.17 (1.01–1.36)*	1.28 (1.09–1.50)**	1.16 (1.04–1.28)**
Adjusted	1.40 (1.20–1.63)**	1.54 (1.30–1.81)**	1.46 (1.31–1.64)**
Low HDL-C			
Crude	1.74 (1.53–1.98)**	1.22 (1.03–1.45)*	1.32 (1.19–1.46)**
Adjusted	1.78 (1.56–2.03)**	1.52 (1.27–1.81)**	1.65 (1.48–1.84)**
High LDL-C			
Crude	1.05 (0.92–1.20)	0.71 (0.61–0.83)**	0.82 (0.74–0.90)**
Adjusted	1.31 (1.15–1.50)**	0.94 (0.80–1.10)	1.12 (1.01–1.25)*

Shown are odds ratios with their 95% confidence intervals in parentheses. Age and history of regular exercise were used as other explanatory variables. In addition, habit of alcohol drinking was also used as an explanatory variable in analysis of overall participants (non, occasional and regular drinkers) and drinking participants (occasional and regular drinkers). WC, waist circumference; WHtR, ratio of waist circumference to height; HDL-C, HDL cholesterol; LDL-C, LDL cholesterol. Symbols denote significant differences (*, *p* < 0.05; **, *p* < 0.01) and marginally significant differences (#, *p* < 0.069; ##, *p* = 0.067) from the reference level of 1.00

### Odds ratios of smokers vs. nonsmokers for high levels of each lipid-related index

The results of multivariate logistic regression analysis for the relationships of smoking with high levels of each lipid-related index are shown in Table 3. In overall participants, nondrinking participants and drinking participants, adjusted odds ratios of smokers vs. nonsmokers for high LDL-C/HDL-C, high TG/HDL-C, high LAP or high CMI were significantly higher than the reference level.

**Table 3:** Odds ratios of smokers vs. nonsmokers for high lipid-related indices in nondrinking participants, drinking participants and overall participants

Variables	Nondrinkers (*n* = 11,408)	Drinkers (*n* = 7385)	Overall participants (*n* = 18,793)
High LDL-C/HDL-C			
Crude	1.93 (1.53–2.43)**	1.47 (1.02–2.11)*	1.51 (1.24–1.83)**
Adjusted	2.29 (1.80–2.91)**	1.96 (1.35–2.85)**	2.17 (1.78–2.66)**
High TG/HDL-C			
Crude	1.44 (1.20–1.74)**	1.47 (1.02–2.11)*	1.30 (1.13–1.50)**
Adjusted	1.68 (1.39–2.02)**	1.76 (1.39–2.21)**	1.70 (1.47–1.97)**
High LAP			
Crude	0.99 (0.89–1.11)	1.42 (1.13–1.77)**	0.92 (0.85–0.99)*
Adjusted	1.21 (1.08–1.36)**	1.13 (1.01–1.27)*	1.17 (1.08–1.27)**
High CMI			
Crude	1.20 (1.08–1.34)**	0.94 (0.85–1.05)	1.06 (0.98–1.15)
Adjusted	1.46 (1.30–1.63)**	1.38 (1.22–1.55)**	1.41 (1.30–1.53)**

Shown are odds ratios with their 95% confidence intervals in parentheses. Age and history of regular exercise were used as other explanatory variables. In addition, habit of alcohol drinking was also used as an explanatory variable in analysis of overall participants (non, occasional and regular drinkers) and drinking participants (occasional and regular drinkers). LDL-C/HDL-C, ratio of LDL cholesterol to HDL cholesterol; TG/HDL-C, ratio of triglycerides to HDL cholesterol. Asterisks denote significant differences from the reference level of 1.00 (*, *p* < 0.05; **, *p* < 0.01)

### Interaction analysis for the relationship between smoking and each lipid variable

Interaction analysis was performed using an interaction term consisting of smoking (nonsmokers vs. smokers) and alcohol drinking (nondrinkers vs. drinkers). As shown in Table 4, the odds ratios of the interaction term for large waist circumference, high WHtR, high triglycerides, low HDL cholesterol, high LDL cholesterol, high LDL-C/HDL-C ratio, high TG/HDL-C ratio, high LAP, and high CMI were not significantly different from the reference level. Only the odds ratio of the interaction term for high LDL cholesterol showed a significant difference from the reference level.

**Table 4:** Odds ratios of smokers vs. nonsmokers for high lipid-related variables in interaction analysis and sensitivity analysis

	Interaction analysis	Sensitivity analysis
Large WC	0.92 (0.72–1.18)	1.04 (0.87–1.23)
High WHtR	1.00 (0.85–1.17)	0.90 (0.80–1.01)
High triglycerides	1.05 (0.85–1.31)	1.40 (1.20–1.64)**
Low HDL-C	0.86 (0.69–1.07)	1.73 (1.51–1.98)**
High LDL-C	0.71 (0.57–0.87)**	1.29 (1.12–1.48)**
High LDL-C/HDL-C	0.88 (0.57–1.36)	2.19 (1.71–2.80)**
High TG/HDL-C	1.00 (0.75–1.34)	1.68 (1.39–2.04)**
High LAP	0.92 (0.79–1.09)	1.21 (1.07–1.36)**
High CMI	0.94 (0.80–1.10)	1.45 (1.29–1.63)**

Shown are odds ratios with their 95% confidence intervals in parentheses. Age and history of regular exercise were used as other explanatory variables. In interaction analysis, the interaction term consisting of smoking and alcohol drinking was added to the explanatory variables, and odds ratios of the interaction term were estimated in overall participants. In sensitivity analysis, odds ratios of light smokers vs. nonsmokers were estimated in nondrinker subjects. WC, waist circumference; WHtR, ratio of waist circumference to height; HDL-C, HDL cholesterol; LDL-C, LDL cholesterol; LDL-C/HDL-C, ratio of LDL cholesterol to HDL cholesterol; TG/HDL-C, ratio of triglycerides to HDL cholesterol. Asterisks denote significant differences from the reference level of 1.00 (**, *p* < 0.01)

### Sensitivity analysis for the relationship between smoking and each lipid variable

In the analysis of participants excluding drinkers and heavy smokers (comparison between light smokers and nonsmokers in nondrinkers), odds ratios of light smokers vs. nonsmokers for high triglycerides, low HDL cholesterol, high LDL cholesterol, high LDL-C/HDL-C ratio, high TG/HDL-C ratio, high LAP, and high CMI were significantly higher than the reference level, while the odds ratios for large waist circumference and high WHtR were not significantly different from the reference level (Table 4). These results are similar to the results of the primary analysis (Tables 2 and 3), and we confirmed the finding of an alcohol-independent association of light smoking with dyslipidemia in Japanese women.

### Comparison of mean levels of each variable comprising lipid-related indices in smokers and nonsmokers

Mean levels of each variable comprising lipid-related indices in overall participants, nondrinking participants and drinking participants were compared in smokers and nonsmokers by using ANCOVA (Table 5). In overall participants, waist circumference was slightly but significantly lower in smokers than in nonsmokers, while in nondrinkers or drinkers, it was not significantly different between nonsmokers and smokers. In overall participants, nondrinking participants and drinking participants, WHtR and HDL cholesterol were significantly lower in smokers than in nonsmokers, and log-transformed triglycerides was significantly higher in smokers than in nonsmokers. In nondrinkers, LDL cholesterol was significantly higher in smokers than in nonsmokers, while in drinkers, LDL cholesterol was significantly lower in smokers than in nonsmokers. In overall participants, LDL cholesterol was not significantly different in nonsmokers and smokers.

**Table 5:** Comparison of mean levels of the variables comprising lipid-related indices between the nonsmoker and smoker groups in nondrinking participants, drinking participants and overall participants

Variables	Nondrinkers (*n* = 11,408)	Drinkers (*n* = 7385)	Overall participants (*n* = 18,793)
WC (cm)			
Nonsmokers	78.3 ± 0.1	77.7 ± 0.1	78.1 ± 0.1
Smokers	78.1 ± 0.3	77.2 ± 0.2	77.7 ± 0.2*
WHtR			
Nonsmokers	0.503 ± 0.001	0.496 ± 0.001	0.500 ± 0.000
Smokers	0.499 ± 0.002*	0.493 ± 0.001*	0.497 ± 0.001**
Log(TG [mg/dl])			
Nonsmokers	1.916 ± 0.002	1.877 ± 0.003	1.901 ± 0.002
Smokers	1.964 ± 0.006**	1.925 ± 0.005**	1.949 ± 0.004**
HDL-C (mg/dl)			
Nonsmokers	63.9 ± 0.1	68.9 ± 0.2	65.8 ± 0.1
Smokers	59.1 ± 0.4**	67.0 ± 0.4**	62.7 ± 0.3**
LDL-C (mg/dl)			
Nonsmokers	117.2 ± 0.3	110.1 ± 0.4	114.4 ± 0.2
Smokers	120.6 ± 0.7**	108.1 ± 0.7*	114.9 ± 0.5

Means ± standard errors of each variable are shown. Age and habit of regular exercise were used as other explanatory variables in ANCOVA. In addition, habit of alcohol drinking was added to the explanatory variables for analysis of overall participants (non, occasional and regular drinkers) and drinking participants (occasional and regular drinkers). WC, waist circumference; WHtR, ratio of waist circumference to height; TG, triglycerides; HDL-C, HDL cholesterol; LDL-C, LDL cholesterol. Asterisks denote significant differences from nonsmokers. *, *p* < 0.05; **, *p* < 0.01

### Comparison of mean levels of each lipid-related index in smokers and nonsmokers

Table 6 shows mean levels of each lipid-related index compared between smokers and nonsmokers by using ANCOVA. In overall participants, nondrinking participants and drinking participants, LDL-C/HDL-C, TG/HDL-C, LAP and CMI were significantly higher in smokers than in nonsmokers.

**Table 6:** Comparison of mean levels of each lipid-related index between the nonsmoker and smoker groups in nondrinking participants, drinking participants and overall participants

Variables	Nondrinkers (*n* = 11,408)	Drinkers (*n* = 7385)	Overall subjects (*n* = 18,793)
LDL-C/HDL-C			
Nonsmokers	1.942 ± 0.007	1.698 ± 0.009	1.845 ± 0.006
Smokers	2.160 ± 0.019**	1.735 ± 0.015*	1.966 ± 0.012**
Log(TG/HDL-C)			
Nonsmokers	0.122 ± 0.003	0.050 ± 0.004	0.093 ± 0.002
Smokers	0.203 ± 0.007**	0.111 ± 0.007**	0.164 ± 0.005**
Log(LAP)			
Nonsmokers	1.221 ± 0.004	1.171 ± 0.005	1.201 ± 0.003
Smokers	1.253 ± 0.010**	1.203 ± 0.009**	1.234 ± 0.007**
Log(CMI)			
Nonsmokers	− 0.180 ± 0.003	− 0.258 ± 0.004	− 0.211 ± 0.002
Smokers	− 0.103 ± 0.008**	− 0.200 ± 0.007**	− 0.143 ± 0.005**

Means ± standard errors of each variable are shown. Age and habit of regular exercise were used as other explanatory variables in ANCOVA. In addition, habit of alcohol drinking was added to the explanatory variables for analysis of overall participants (non, occasional and regular drinkers) and drinking participants (occasional and regular drinkers). LDL-C/HDL-C, ratio of LDL cholesterol to HDL cholesterol; TG/HDL-C, ratio of triglycerides to HDL cholesterol. Asterisks denote significant differences from nonsmokers. *, *p* < 0.05; **, *p* < 0.01

### Comparison of mean levels of each variable comprising lipid-related indices or each lipid-related index in nondrinkers and drinkers of the nonsmoking participant group

In order to investigate the relationships of drinking with each variable, mean levels of each variable in nonsmoking participants were compared among nondrinkers, occasional drinkers and regular drinkers by using ANCOVA (Table 7). WHtR was significantly lower in regular drinkers than in nondrinkers and occasional drinkers, while waist circumference was not significantly different among non-, occasional and regular drinkers. HDL cholesterol and LDL cholesterol were significantly higher and lower, respectively, in occasional drinkers than in nondrinkers and were significantly higher and lower, respectively, in regular drinkers than in nondrinkers and occasional drinkers. Log-transformed triglycerides was significantly lower in occasional and regular drinkers than in nondrinkers. LDL-C/HDL-C, TG/HDL-C and CMI were significantly lower in occasional drinkers than in nondrinkers and were significantly lower in regular drinkers than in nondrinkers and occasional drinkers. LAP was significantly lower in regular drinkers than in nondrinkers but was not significantly different in occasional drinkers and nondrinkers.

**Table 7:** Comparison of mean levels of each lipid-related index or each variable comprising lipid-related indices in nondrinkers and drinkers of the nonsmoker group

	Nondrinkers (*n* = 11,408)	Occasional drinkers (*n* = 5609)	Regular drinkers (*n* = 1776)
WC (cm)	78.3 ± 0.1	78.2 ± 0.1	77.6 ± 0.3
WHtR	0.503 ± 0.001	0.501 ± 0.001	0.496 ± 0.002**,†
Log(TG [mg/dl])	1.914 ± 0.002	1.895 ± 0.003**	1.885 ± 0.007**
HDL-C (mg/dl)	63.9 ± 0.1	67.2 ± 0.2**	74.2 ± 0.4**,††
LDL-C (mg/dl)	116.8 ± 0.3	114.0 ± 0.4**	107.0 ± 0.9**,††
LDL-C/HDL-C	1.936 ± 0.007	1.800 ± 0.010**	1.529 ± 0.021**,††
TG/HDL-C	0.119 ± 0.003	0.078 ± 0.004**	0.025 ± 0.009**,††
LAP	1.217 ± 0.004	1.201 ± 0.006	1.179 ± 0.012**
CMI	− 0.183 ± 0.003	− 0.225 ± 0.005**	− 0.283 ± 0.009**,††

Means ± standard errors of each variable are shown. Age and habit of regular exercise were used as other explanatory variables in ANCOVA. WC, waist circumference; WHtR, ratio of waist circumference to height; TG. triglycerides; HDL-C, HDL cholesterol; LDL-C, LDL cholesterol; LDL-C/HDL-C, ratio of LDL cholesterol to HDL cholesterol; TG/HDL-C, ratio of triglycerides to HDL cholesterol. Symbols denote significant differences from nondrinkers (**, *p* < 0.01) and occasional drinkers (†, *p* < 0.05; ††, *p* < 0.01)

## Discussion

In women, smoking was positively associated with triglycerides and lipid-related indices including LDL-C/HDL-C, TG/HDL-C, LAP and CMI, while there was an inverse association of smoking with HDL cholesterol. These relationships of smoking were independent of alcohol drinking (Table 8), although the percentages of occasional and regular drinkers were significantly higher in smokers than in nonsmokers (Table 1). On the other hand, the relationship between smoking and LDL cholesterol was potently influenced by alcohol drinking: Smoking showed a positive association with LDL cholesterol in nondrinkers but not in drinkers (Table 8), and a significant interaction of alcohol drinking with smoking was found for high LDL cholesterol (Table 4). Therefore, alcohol drinking confounded the relationship of smoking with LDL cholesterol but not the relationships of smoking with other lipid-related variables tested. To the best of our knowledge, this study is the first study showing alcohol-independent associations of light smoking with lipid profiles and lipid-related indices in Asian women and the results will be useful for prevention of cardiovascular disease from the viewpoint of public health.

**Table 8:** Summary of the results obtained by analyses (ANCOVA and logistic regression analysis) for the relationships of smoking with lipid-related indices and the variables comprising these indices

Variables	Nondrinkers		Drinkers		Overall subjects	
	A	L	A	L	A	L
WC	**→**	**→**	**→**	**→**	**↓**	**→**
WHtR	**↓**	**↓**	**↓**	**↓**	**↓**	**↓**
Triglycerides	**↑**	**↑**	**↑**	**↑**	**↑**	**↑**
HDL-C	**↓**	**↓** *	**↓**	**↓** *	**↓**	**↓** *
LDL-C	**↑**	**↑**	**↓**	**→**	**→**	**↑**
LDL-C/HDL-C	**↑**	**↑**	**↑**	**↑**	**↑**	**↑**
TG/HDL-C	**↑**	**↑**	**↑**	**↑**	**↑**	**↑**
LAP	**↑**	**↑**	**↑**	**↑**	**↑**	**↑**
CMI	**↑**	**↑**	**↑**	**↑**	**↑**	**↑**

Results of ANCOVA (**A**, comparison of means of each variable between nonsmokers and smokers) and logistic regression analysis (**L**, comparison of frequencies of abnormality in each variable between nonsmokers and smokers) are shown in the left and right sides, respectively, of each cell in the table.**↑**, higher in smokers than in nonsmokers; **↓**, lower in smokers than in nonsmokers; **→**, not different in smokers and nonsmokers. *: “**↓**” here indicates higher frequency of hypo-HDL cholesterolemia. WC, waist circumference; WHtR, waist-to-height ratio; HDL-C, HDL cholesterol; LDL-C, LDL cholesterol; LDL-C/HDL-C, ratio of LDL cholesterol to HDL cholesterol; TG/HDL-C, ratio of triglycerides to HDL cholesterol

There is a strong association between frequencies of smokers and drinkers [20]. As shown in Table 7, the associations of alcohol drinking with blood lipids and lipid-related indices were opposite to the associations of smoking with them: HDL cholesterol was higher and triglycerides, LDL cholesterol and all of the four lipid indices were lower in drinkers than in nondrinkers. Thus, alcohol drinking should be taken into account when the relations of smoking with lipid-related indices are considered. In nondrinkers, smoking was positively associated with triglycerides and LDL cholesterol, was inversely associated with HDL cholesterol and WHtR, and was not significantly related to waist circumference. Therefore, alcohol-independent positive associations of smoking with the four lipid-related indices in women results from proneness of smokers to dyslipidemia, such as hyper-triglyceridemia, hypo-HDL cholesterolemia and hyper-LDL cholesterolemia. This is the first study showing the associations between smoking and recent lipid-related indices and clarifying the reasons for the associations in women.

It is needless to say that smoking should be avoided from the viewpoint of prevention of cardiovascular disease. The lipid-related indices are discriminators for cardiovascular disease, and consistent positive associations between smoking and the lipid indices in women were found in this study. Therefore, smoking facilitates progression of atherosclerosis through deterioration of the blood lipid profile, resulting in increased risk of cardiovascular disease. In a recent study in which database of Chinese male subjects was analyzed, smoking was associated with TG/HDL-C and LAP independently of alcohol drinking [29]. In a study using Japanese male participants aged 35–60 years [30], there was a positive dose-dependent association between smoking and high CMI: odds ratios vs. nonsmokers for high CMI were 1.16 (95% confidence interval [CI]: 1.10–1.23) in light smokers (≤ 20 cigarettes per day), 1.60 (95% CI: 1.49–1.70) in heavy smokers (> 20 and ≤ 40 cigarettes per day) and 2.34 (95% CI: 1.77–3.09) in very heavy smokers (> 40 cigarettes per day). In the present study using Japanese female participants, the odds ratio was 1.41 (95% CI: 1.30–1.53) in overall smokers (Table 3), most of whom (99.2%) were light smokers. After excluding heavy smokers (0.08%), the odds ratio in light smokers vs. nonsmokers for high CMI was calculated to be 1.40 (95% CI: 1.28–1.52, *p* < 0.01) in the present study. Thus, the odds ratio in light smokers was higher in women than in men, suggesting a stronger association between smoking and CMI in women than in men. This agrees with the results of a previous systematic review and meta-analysis study showing that the detrimental effect of smoking was stronger for women than for men in ischemic heart disease [19]. Interestingly, the above odds ratio in light smokers vs. nonsmokers for high CMI in women was 1.21-fold higher than the odds ratio in men, and this ratio is close to the female-to-male relative risk ratio of smoking for coronary heart disease of 1.25 shown by the above meta-analysis study [19]. Therefore, smoking shows a detrimental effect on cardiometabolic health in women, although the amount of smoking is much less in female smokers than in male smokers.

There are limitations of this study. Although blood lipid levels are influenced by menopause, no information on menopause was available for the participants of this study. Age and habits of alcohol drinking and regular exercise were adjusted in the multivariate analyses. However, there are other possible confounders for the relationships between smoking and blood lipid-related indices, including diet, education, occupation and socio-economic status, for which information was not available in this study. The participants of this study were Japanese women, and further studies using female participants with other races and/or ethnicities are needed to confirm the findings of this study. Since the design of this study is cross-sectional, causality of the associations between smoking and lipid-related indices cannot be discussed.

## Conclusions

Smoking is positively associated with lipid-related indices in women independently of alcohol drinking (Table 8), although drinkers are much more frequent in smokers than in nonsmokers. High lipid-related indices in smokers reflect their proneness to dyslipidemia such as hypertriglyceridemia, hyper-LDL-cholesterolemia and hypo-HDL-cholesterolemia.

## Abbreviations

ANCOVA: Analysis of covariance
CETP: Cholesterol ester transfer protein
CMI: Cardio metabolic index
HDL-C: High density lipoprotein cholesterol
LAP: Lipid accumulation product
LCAT: Lecithin cholesterol acyltransferase
LDL-C: Low density lipoprotein cholesterol
LDL-C/HDL-C: The ratio of LDL cholesterol to HDL cholesterol
LPL: Lipoprotein lipase
TG: Triglycerides
TG/HDL-C: The ratio of triglycerides to HDL cholesterol
WC: Waist circumference
WHtR: The ratio of waist circumference to height
